# Study of *Tnp1*, *Tekt1*, and *Plzf* Genes Expression During an *in vitro* Three-Dimensional Neonatal Male Mice Testis Culture

**DOI:** 10.22034/ibj.22.4.258

**Published:** 2018-07

**Authors:** Ahmad Alrahel, Mansoureh Movahedin, Zohre Mazaheri, Fardin Amidi

**Affiliations:** 1 Anatomical Sciences Department, Faculty of Medical Sciences, Tarbiat Modares University, Tehran, Iran; 2 Anatomical Sciences Department, Faculty of Medicine, Tehran University of Medical Sciences, Tehran, Iran

**Keywords:** Culture, Mice, Testis

## Abstract

**Background:**

*In vitro* spermatogenesis has a long research history beginning in the early 20^th^ century. This organ culture method was therefore abandoned, and alternative cell culture methods were chosen by many researchers. Here, whether *Tnp1*, *Tekt1*, and *Plzf*, which play a crucial role in spermatogenesis, can be expressed during testis organ culture was assessed.

**Methods:**

Testes of 10 mouse pups were first removed, and the testis tissue was then separated into smaller pieces of seminiferous tubules. The size of the pieces was arbitrary; approximately 1 mg in weight or 1 mm^3^ in size when compacted. Afterwards, the testis tissue fragments (1–3) were transferred to the hexahedrons, incubated in a culture incubator and cultured for 12 weeks. Histological assessment and molecular evaluation were carried out at the end of the study.

**Results:**

The results showed that the expression of *Tekt1* as a mitotic gene in mouse pups decreased significantly (*p* ≤ 0.05) in comparison to adult mouse testis. Meanwhile, the expression of *Tnp1* as a meiotic gene increased significantly (*p* ≤ 0.05) as compared to neonate mouse testis at the beginning of the culture. The expression of *Plzf* showed no significant difference during the 12 weeks of culture (*p* ≥ 0.05). Based on histological study, different types of spermatocytes and post-meiotic stages of germ cells could not be detected.

**Conclusion:**

This kind of three-dimensional culture can induce expression of post-meiotic gene, *Tnp1*, but only at the molecular level and not beyond meiosis.

## INTRODUCTION

The history of *in vitro* spermatogenesis dates back to the early 20^th^ century[1,2]. This process did not proceed beyond the meiotic pachytene stage. Therefore, this organ culture method was neglected by many researchers, and some other cell culture methods were selected. Nonetheless, it remained impossible to induce a complete spermatogenesis process from spermatogonia or from spermatognial stem cells *in vitro* in a reliable manner.

In the 1980s, organ culture methods were replaced with cell culture methods, which were used for *in vitro* spermatogenesis. Although previous investigations have reported the completion of meiosis or spermatogenesis *in vitro*, this issue remains a challenge, which hinders the production of haploid cells that are proven to be fertile from spermatogonial stem cells under the cell culture conditions[3,4]. Furthermore, little is known about the molecular mechanisms of self-renewal and differentiation of spermatogonia and mitotic germ cells of the testis into sperm.

Mammalian spermatogenesis is characterized by significant changes in the chromatin structure and the replacement of nucleoproteins. In elongating and condensing spermatids, the major chromatin structure is restructured, where the histones are first replaced by transition proteins (TPs), which are in turn replaced by protamines[5]. TP1 and TP2, which are encoded by *Tnp1* and *Tnp2* genes, respectively, were found to be the major TPs in rodent spermatids. Both are basic proteins rich in arginine and lysine and bind DNA strongly [6]. These TPs are expressed exclusively in post-meiotic, haploid spermatids. In mice, the first detection of *Tnp1* and *Tnp2* mRNAs occurred in step 7 round spermatids, and their degradation was observed at steps 13 and 14[7,8].

The protein TEKT also contributes to cilia, flagella, basal bodies, and centrioles. In mammals, five TEKTs (TEKT1, 2, 3, 4, and 5) have been identified in testis and spermatozoa[9,10]. These proteins, except TEKT1, have been reported to be present in spermatozoa, predominantly localized at the peri-axoneme structures of the flagella, i.e. mitochondria and outer dense fibers[11]. In addition to *Tnp1* and *Tekt1*, the essential role of *Plzf* in the maintenance of spermatogonial stem cells has been revealed previously[12]. The study of these proteins and those involved in spermatogenesis is very important to the concept of *in vitro* spermatogenesis[13]. In the present study, we attempted to assess whether *Tnp1*, *Tekt1*, and *Plzf*, which have essential roles in spermatogenesis, can be expressed during organ culture of testis. To accomplish this, organ culture of neonatal male mice was used to understand spermatogenesis development at molecular level for the course of 12 weeks.

## MATERIALS AND METHODS

### Media

For the preparation of organ culture medium (α-minimum essential medium [α-MEM] + 10% knockout serum replacement [KSR]), 10.1 g α-MEM was dissolved in 500 ml of double-deionized water (DDW) to make a double-concentrated α-MEM. Subsequently, 20 ml of KSR, 2 ml of penicillin-streptomycin, and 5.2 ml of sodium bicarbonate (7%) were added to 100 ml of double-concentrated α-MEM. Distilled water was then added to the mixture so as to achieve a total volume of 200 ml. The culture medium was sterilized by its filtering through a 0.22-µm Millipore membrane filter and stored in a refrigerator (2–8 °C).

### Agarose dish preparation

Agarose (1.5 g) was mixed with 100 ml of DDW in a 200-ml flask and then gently shaken to suspend the agarose. The mixture was then sterilized by autoclaving. Agarose solution (33 ml) was then poured into three 10-cm dishes, where the depth of the solution was about 5 mm. These solutionsswere left for about 2 h at room temperature until the agarose gels become solid. These prepared dishes can be preserved by sealing and storing in a refrigerator for several weeks. Three to four hexahedrons were placed into each well of a six-well plate. To soak the gels completely, culture medium (α-MEM + 10% KSR) was added. The gels were kept in an incubator overnight or longer in order to allow the replacement of water in the agarose with the medium. At the initiation of the culture experiment, the old medium was removed by aspiration, and a fresh medium was added to the well, between the half and the four-fifth height of the agarose gel pieces[15,16].

### Sampling

### Preparing neonatal mouse testicular tissue

This study was approved by the Ethics Committee of Tarbiat Modares University with the Ref. number: 52/3080 and Date: 27/07/2014. After euthanizing the mouse pups, the testes were removed from 10 NMRI neonatal male mice (six days old) and placed immediately in 3.5-cm dishes containing the culture medium. The tunica albuginea was held with two fine forceps at two close sites and then pulled apart to tear the tunica, thus exposing the seminiferous tubules inside. The tunica was removed after fully exposing the seminiferous tubules. Subsequently, a sufficient quantity of seminiferous tubules was collected into a dish containing fresh culture medium, cooled on ice. The testis tissue was separated into smaller pieces of seminiferous tubules using forceps. The size of the pieces was arbitrary; approximately 1 mg in weight or 1 mm^3^ in size when compacted. The pieces were maintained in the culture medium, and each tissue fragment was held gently using forceps or held with a micropipette by applying mild negative pressure[17]. One up to the three testis tissue fragments was transferred to the hexahedrons and then incubated by placing the six-well plates in a culture incubator. The culture incubator was supplied with 5% CO_2_ in the air and maintained at 34 °C. The medium was changed twice a week by removing the old medium in a well through aspiration, and then the same amount of fresh culture medium was added.

### Real-time PCR studies: quantitative analysis of gene expression

The total RNA was extracted from five samples for every group of neonatal mouse and fresh tissue from 12 weeks of three-dimensional cultured tissue using RNX-Plus™ (CinnaGen, Iran), as per the manufacturer’s recommendations. Genomic contamination was eliminated by treating the RNA with DNase I (Fermentas, Germany). Concentrations of RNA samples were determined by UV spectrophotometry (Eppendorf Company, USA). cDNAs synthesis was performed using RevertAid™ First Strand cDNA Synthesis kit (Fermentas, Germany) and oligo (dT) primers. For PCR reactions, primers were adapted from other primers (designed by the NCBI website (https://www.ncbi.nlm.nih.gov/)[18-20] and synthesized by a commercial source (CinnaGen, Iran; Table 1). The PCRs were performed using Master Mix and SYBR Green I by Applied Biosystems, StepOne™ thermal cycler (Applied Biosystems, USA). The PCR program was initiated by a melting cycle of 5 min at 95 °C. This step was followed by 40 cycles of melting (30 s at 95 °C), annealing (30 s at 58 °C), and extension (30 s at 72 °C). The quality of the PCR reactions was confirmed by the melting curve analyses. A standard curve was used to determine the efficiency for each gene (logarithmic dilution series of cDNA from the testes). The reference (β-actin) and the target genes for each gene were amplified in the same run. In addition, this process was repeated and duplicated three times for all the target and reference genes. The reference genes were approximately equal, and the target genes were normalized to the reference gene and expressed relative to a calibrator (12 weeks, three-dimensional cultured tissue).

**Table 1:** Primers used for qRT-PCR

Genes	Primer sequence	Length (bp)
*Tekt1*	F:5’GCTGGCTGAACATCTGG 3’ R:5’TTCTTGCTGCGTGATGGC 3’	91
*Tnp1*	F:5’TGTGATGCGGCAATGAGC 3’ R:5’CGACTGGGATTTACCCACTC 3’	142
*Plzf*	F:5’GCTGCTGTCTCTGTGATGG 3’ R:5’GGGCTGATGGAACATAGGGG 3’	154
*β-actin*	F:5’TTACTGAGCTGCGTTTTACAC3’ R:5’ACAAAGCCATGCCAATGTTG3’	90

### Histological evaluation

The specimens were fixed with Bouinns wiis and embedded in paraffin. Thin sections were made in the horizontal direction to obtain the largest cut surface.

### Statistical analysis

The data were analyzed using one-way repeated measure analysis of variance (ANOVA), followed by Tokay’s post hoc test and were then presented as mean ± SD (standard deviation). The calculations were performed using the statistics software package SPSS Version 18.0. The results were deemed to be significant only when *p* ≤ 0.05. For real-time PCR data, the logarithmic values were converted to the real values by raising two to the power of the ΔΔCt value before statistical analysis..

## RESULTS

### Organ culture

**Fig. 1:**
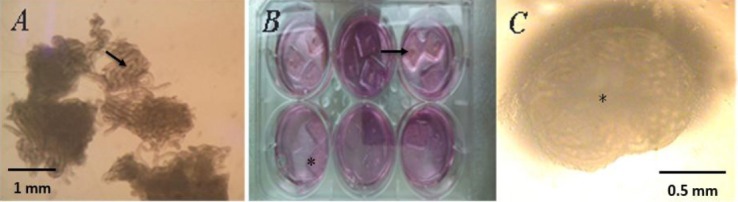
Preparation of mouse neonatal testis. (A) Dissociation of the testis tissue into fragments (they were approximately 1 mm^3^ in size when compacted) suitable for tissue culture. Arrow shows the seminiferous tubules. (B) Testes tissue pieces put on the agarose gel in three-dimensional organ culture method; agarose gel is shown by *, and fragment tissue is indicated by arrow. (C) Photomicrograph of tissue sections following a 12-week culture; the dark part in the center of tissue section represents limited access to nutrition (*).

Neonatal mouse testicular tissue was cultured in α-MEM + 10% KSR for 12 weeks (Fig. 1A). For organ culture, agarose gel of 1.5% (w/v) was adopted for all the experiments. The amount of medium was adjusted so that its surface level was just below the upper surface of the agarose gel (Fig. 1B). The medium covered the agarose gel entirely, thereby submerging the tissues, which led to the tissues being compacted and necrotic. The results showed that after preparation of agarose for the culture of testicular tissue components, these components could be used to culture for 12 weeks without any changes, such as darkness at the center, which is a marker for necrosis or apoptosis. This result was observed after creating *in vitro* conditions; 4/5 of agarose environment was put in the culture environment, and the samples continued their growth without floating in the environment or the culture medium. The results obtained from the macroscopic examination of seminiferous tubules from the cultured testicular tissues after 12 weeks showed no changes in the tubules anatomical structure, such as disruption in the integrity of the tubules or interstitial tissues (Fig. 1C).

**Fig. 2:**
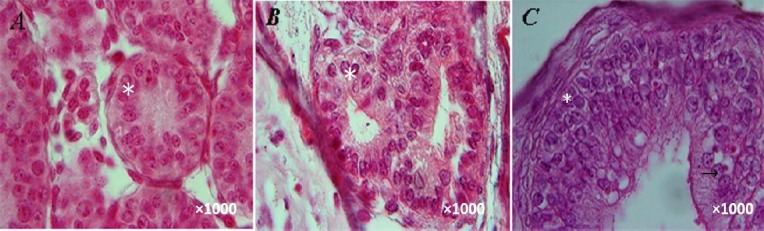
Histological section of the testis tissue fragment cultured for (A) 8 weeks, (B) 10 weeks, and (C) 12 weeks. Stars and the arrow demonstrate spermatogonial cell and spermatocyte cell during culture, respectively.

### Histological assessment

Examination of the tissue pieces showed that spermatogonial stem cells were present in the seminiferous tubules of neonatal male mouse testicular tissues, before and after the three-dimensional culture from the basal to the center of the tubule. In addition, it was observed that the outer diameter of the tubules was approximately 50–60 micrometers. On the other hand, there was an absence of lumen in the central space of the tubules. Leydig cells were also present in the interstitial space. These observations were investigated during weeks 8 (Fig. 2A), 10 (Fig. 2B), and 12 (Fig. 2C). The results showed that in the cultured pieces of seminiferous tubules, which were similar to the culture medium, the outer diameter of tubules was about 100–120 micrometers during weeks 8, 10, and 12. In addition, the lumen was investigated at different times. However, among all the cell levels, only spermatogonial level was observed at weeks 8 and 10. The results of these cell level studies showed that 12- week culture can create spermatocyte cell levels in seminiferous tubules. It should be noted that tissue integrity was preserved in all the groups, and Sertoli cells and blood-testis barriers were in their places. At the same time, tubules were not observed at the center of tissue section, which died due to hypoxic conditions and limited access to nutrition (Fig. 2C).

### Molecular assessment

**Fig. 3:**
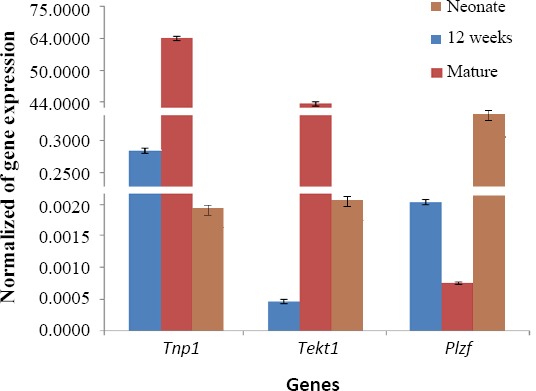
Expression mitotic and meiotic genes *Tnp1*, *Tekt1*, and *Plzf*. 12 weeks, testis tissue fragment that cultured for 12 weeks; mature, testis tissue from mature mouse. Data were normalized to β-actin and represented mean ± SE after three times repeats. There are significant differences between the groups in each gene (*p* < 0.05).

The expression of germ-cells specific genes, 12 weeks after the three-dimensional culture of neonatal mice testis, showed that the *Plzf* gene expression increased significantly (*p* ≤ 0.05) when compared to the adult tissue (the same amount of tissue was cultured). Also, our results showed that this level was significantly lower (*p* ≤ 0.05) in the 12-week three-dimensional culture of neonatal male mice testis as compared to fresh neonatal male testis. Additionally, the results indicated that the spermatocyte cell levels reduced significantly (*p* ≤ 0.05) in neonatal testis after the 12 weeks of culture in comparison to the mature male mice testicular tissue. Furthermore, the level of expression decreased for spermatocytes in the three-dimensional culture group as compared to the fresh neonatal and mature mice. The spermatocytes population was high (*p* ≤ 0.05) in mature testis in comparison to the fresh neonatal testis (Fig. 3).

According to the results, *Tnp1* expression in cultured tissue was greater than all the other categories. This increase showed a significant difference when compared to the mature group (*p* ≤ 0.05). Based on the data, spermatid gene marker elevated significantly (*p* ≤ 0.05) after 12 weeks of culture as compared to the fresh neonatal male mice testis. The gene expression of *Tnp1*, however, was highest in the tissue mature male testis.

## DISCUSSION

Today, three-dimensional culture is considered as a very useful technique in the preservation and development of a variety of tissues, such as germinal tissues. Thus, in this study, agarose gel and culture medium (α-MEM + 10% KSR) were used for the three-dimensional culture of immature mouse testicular tissue. Germ cell molecular changes are very valuable tools for improving spermatogenesis. The results obtained from this study revealed that the use of “α-MEM by KSR” can cause tissue survival and also can improve the spermatogenesis process during a 12-week culture. The Gohbara *et al*.’s[21] study includes 2 mM of glutamine and 0.28 mM of vitamin C. DMEM also contains 4 mM glutamine. As all the media that were used included 10% FBS, comparing the detailed substances of the media and the supplements added to them with those of the Steinberger’s *et al*. study[22] was deemed worthless. Future studies should omit FBS and replace it with chemically defined supplements in order to better understand the environmental factors that influence spermatogenesis[23]. In a previous study, Sato *et al*.[16] found that serum-free medium, supplemented with KSR, is suitable for inducing spermatogenesis from neonatal mouse testes. This environment was able to cause an increase in the lumen diameter, maintain the integrity of testicular tissue in tissue sections, and create spermatogonial cells and spermatocytes. In the Gohbara’s[21] study, the penetration of O_2_ into the tissues was improved (shown by the smaller area of central necrosis in each tissue), the seminiferous tubules distortion increased, and the number of spermatogonia and spermatocytes decreased dramatically. On the other hand, histological examination alone seemed insufficient to evaluate the progress of meiosis accurately. They were also not able to find spermatids by using histology[21]. The histological evaluation showed only two classes of spermatogonia and spermatocyte. However, molecular studies showed that there is a meaningful increase in the marker specific for meiotic cell gene expression.

In previous studies, progress reports often provided information until the spermatocytes stage. In Yokonishi’s[24] study, however, the presence of spermatid cell and even sperm, which leads to embryo formation, has been reported. Organ culture of testis tissues for spermatogenesis seems to be more natural as the micro-environmental condition for germ cells is close to that of *in vivo*. Spermatogonial cells in the tubes seemed to be able to resume the process of spermatogenesis. However, the level of spermatocytes formed was unstable, and inter-meiosis process occurred immediately after formation. Therefore, the expression of *Tnp1* form, which is considered as a meiotic marker, increased significantly in comparison to *Tekt1* gene, which is considered as a spermatocyte cell specific mitotic marker. Nonetheless, no spermatid level was observed in the tissue studies.

The results of this study, which consists of results related to the neonatal mouse testicular tissue cultured for 12 weeks, histological studies, and the specific genes expression of the germinal cells studies, indicate the significant success of *Tnp1* expression as spermatid gene marker elevated after 12 weeks in cultured tissue. Further studies are needed, where the culture medium enriched with several supplements or growth factors, for better results. In our future researches, the goal is to achieve mouse testicular tissue three-dimensional culture. These results will encourage researchers to set up a culture system for *in vitro* spermatogenesis.
